# Supplementary material for: Neural network analysis of clinical variables predicts escalated care in COVID-19 patients: a retrospective study
Source: PeerJ. 2021 Apr 19;9:e11205. doi: 10.7717/peerj.11205 (PMC8061580; doi:10.7717/peerj.11205)
Supplement: Supplemental Information 1 [file peerj-09-11205-s001.pdf]

Stony Brook Medicine

PUI Registry

Codebook

Data Dictionary Codebook

05/07/2020 3:01pm

Collapse all instruments

	#	Variable / Field Name	Field Label *Field Note*	Field Attributes (Field Type, Validation, Choices, Calculations, etc.)
Instrument: **Demographics** (demographics)				Collapse
	1	record_id	Record ID	text, Required
	2	mrn	MRN	text (number), Required, Identifier
	3	encounternumber	Encounter Number	text (number), Required, Identifier
	4	ethnicity	Ethnicity	radio, Required<br>1 Hispanic/Latino<br>2 Not Hispanic/Latino<br>3 Unknown/Not Reported
	5	race	Race	radio, Required<br>1 Caucasian<br>2 African American<br>3 Asian<br>4 American Indian/Alaska Native<br>5 Native Hawaiian or Other Pacific Islander<br>6 More than One Race<br>7 Unknown/Not Reported
	6	domicile	Domicile	radio, Required<br>1 Private Home<br>2 Assisted Living/Skilled Nursing Facility<br>3 Veterans Home<br>4 Group Home
	7	facility_name<br><br>Show the field ONLY if:<br>[domicile] = '2' or [domicile] = '3' or [domicile] = '4'	What is the name of the facility where the patients live?	notes, Required
	8	demographics_complete	Section Header: *Form Status*<br>Complete?	dropdown<br>0 Incomplete<br>1 Unverified<br>2 Complete
Instrument: **Symptoms At Presentation** (symptoms_at_presentation)				Collapse
	9	date_of_presentation	Date of Presentation	text (date_mdy)
	10	onset_known	Is approximate date of symptom onset known?	yesno, Required<br>1 Yes<br>0 No
	11	date_symptom_onset<br><br>Show the field ONLY if:<br>[onset_known] = '1'	Date of Symptom Onset	text (date_mdy), Required

	12	symptoms	Symptoms (check all that apply)	checkbox, Required<br>1 symptoms___1 Fever (patient reported)<br>2 symptoms___2 Cough<br>3 symptoms___3 SOB<br>4 symptoms___4 Fatigue<br>5 symptoms___5 Sputum<br>6 symptoms___6 Myalgia<br>7 symptoms___7 Diarrhea<br>8 symptoms___8 Nausea or vomiting<br>10 symptoms___10 Sore throat<br>11 symptoms___11 Runny nose/nasal congestion<br>12 symptoms___12 Loss of smell<br>13 symptoms___13 Loss of taste<br>14 symptoms___14 Headache<br>15 symptoms___15 chest discomfort, chest pain<br>9 symptoms___9 Asymptomatic
	13	covid_at_presentation	Is the patient presenting with a known positive COVID 19 test result?	yesno, Required<br>1 Yes<br>0 No
	14	travel_yn	Recent Travel?	yesno, Required<br>1 Yes<br>0 No
	15	travel_where<br>Show the field ONLY if:<br>[travel_yn] = '1'	Where did patient travel?	radio, Required<br>1 China<br>2 South Korea<br>3 Asia (besides China & South Korea)<br>4 Iran<br>5 Italy<br>6 Europe besides Italy<br>7 Other<br>8 Domestic Travel
	16	othertravellocation<br>Show the field ONLY if:<br>[travel_where] = '7'	If Other, please specify:	text
	17	sickcontacts	Sick Contacts?	yesno, Required<br>1 Yes<br>0 No
	18	exposure	Exposure to someone with confirmed COVID-19 infection?	yesno, Required<br>1 Yes<br>0 No
	19	healthcare_worker	Does the patient work in a healthcare facility?	yesno, Required<br>1 Yes<br>0 No
	20	symptoms_at_presentation_complete	Section Header: *Form Status*<br>Complete?	dropdown<br>0 Incomplete<br>1 Unverified<br>2 Complete

Instrument: **Medical History** (medical_history) Collapse

	21	smoking_history	Smoking History	radio, Required<br>1 Current Smoker<br>2 Former Smoker<br>3 Never Smoker<br>4 Unknown
	22	vaping_history	Vaping History?	radio, Required<br>1 Current<br>2 Former<br>3 Never<br>4 Unknown
	23	pregnancy	Is the patient pregnant?	yesno, Required<br>1 Yes<br>0 No
	24	hypertensionhx	History of Hypertension?	yesno, Required<br>1 Yes<br>0 No
	25	diabeteshx	History of Diabetes?	yesno, Required<br>1 Yes<br>0 No
	26	asthmahx	History of Asthma?	yesno, Required<br>1 Yes<br>0 No
	27	coronaryheartdiseasehx	History of coronary heart disease?	yesno, Required<br>1 Yes<br>0 No
	28	copdhx	History of COPD?	yesno, Required<br>1 Yes<br>0 No
	29	heartfailurehx	History of Heart Failure?	yesno, Required<br>1 Yes<br>0 No
	30	carcinomahx	History of carcinoma?	yesno, Required<br>1 Yes<br>0 No
	31	immunosuppressionhx	History of Immunosuppression?	yesno, Required<br>1 Yes<br>0 No
	32	ckdhx	History of Chronic Kidney Disease?	yesno, Required<br>1 Yes<br>0 No
	33	medhxother	Other significant medical history?	yesno, Required<br>1 Yes<br>0 No
	34	othermedhxspecify<br>Show the field ONLY if:<br>[medhxother] = '1'	Please specify:	text, Required

	35	medical_history_complete	Section Header: *Form Status*<br>Complete?	dropdown<br>0 Incomplete<br>1 Unverified<br>2 Complete
Instrument: **Lab Results At Presentation** (lab_results_at_presentation)				Collapse
	36	labs_ordered	Was COVID testing ordered?	checkbox, Required<br>4 labs_ordered___4 Yes (Coronavirus (SARs-Cov-2 by PCR))<br>15 labs_ordered___15 No COVID testing ordered
	37	covid_19_result<br>Show the field ONLY if:<br>[labs_ordered(4)] = '1'	COVID-19 Test Result	radio<br>1 Positive<br>2 Negative<br>3 Indeterminate result
	38	lab_results_at_presentation_complete	Section Header: *Form Status*<br>Complete?	dropdown<br>0 Incomplete<br>1 Unverified<br>2 Complete
Instrument: **Imaging Results At Presentation** (imaging_results_at_presentation)				Collapse
	39	imaging_ordered	Imaging Ordered	checkbox, Required<br>1 imaging_ordered___1 Chest AP Portable<br>2 imaging_ordered___2 CT Chest<br>3 imaging_ordered___3 No Imaging Ordered
	40	chest_xray_result<br>Show the field ONLY if:<br>[imaging_ordered(1)] = '1'	Chest X-Ray Result (only consider infectious findings)	radio<br>1 Positive<br>2 Negative
	41	findings_chest_ap<br>Show the field ONLY if:<br>[chest_xray_result] = '1'	Were positive findings unilateral or bilateral?	radio<br>1 Unilateral<br>2 Bilateral
	42	ct_chest_result<br>Show the field ONLY if:<br>[imaging_ordered(2)] = '1'	CT Chest Result (only consider infectious findings)	radio<br>1 Positive<br>2 Negative
	43	findings_ct_chest<br>Show the field ONLY if:<br>[ct_chest_result] = '1'	Were positive findings unilateral or bilateral?	radio<br>1 Unilateral<br>2 Bilateral
	44	imaging_results_at_presentation_complete	Section Header: *Form Status*<br>Complete?	dropdown<br>0 Incomplete<br>1 Unverified<br>2 Complete
Instrument: **Treatments** (treatments)				Collapse
	45	oxygen	Oxygen Therapy?	yesno<br>1 Yes<br>0 No

	46	oxygentype<br><br>Show the field ONLY if:<br>[oxygen] = '1'	Specify what type of oxygen therapy was provided (check all that apply)	checkbox, Required<br>1 oxygentype___1 nasal cannula<br>2 oxygentype___2 nonrebreather<br>3 oxygentype___3 ventimask<br>4 oxygentype___4 blow-by oxygen<br>5 oxygentype___5 trach collar
	47	invmech_ventilation	Did the patient require invasive mechanical ventilation?	yesno, Required<br>1 Yes<br>0 No
	48	ems_intubation<br><br>Show the field ONLY if:<br>[invmech_ventilation] = '1'	Was the patient intubated in the field by EMS?	yesno, Required<br>1 Yes<br>0 No
	49	ed_intubation<br><br>Show the field ONLY if:<br>[invmech_ventilation] = '1'	Was the patient intubated in the ED?	yesno, Required<br>1 Yes<br>0 No
	50	start_invmech_vent<br><br>Show the field ONLY if:<br>[invmech_ventilation] = '1'	Start time of invasive mechanical ventilation	text (datetime_mdy), Required
	51	stop_invmech_vent<br><br>Show the field ONLY if:<br>[invmech_ventilation] = '1'	Stop Time of Invasive Mechanical Ventilation	text (datetime_mdy), Required
	52	invmech_vent_time<br><br>Show the field ONLY if:<br>[invmech_ventilation] = '1'	Time of Invasive Mechanical Ventilation	calc<br>Calculation: datediff([stop_invmech_vent], [start_invmech_vent],"d","mdy")
	53	re_intubated<br><br>Show the field ONLY if:<br>[invmech_ventilation] = '1'	Was the patient re-intubated during their hospital stay?	yesno, Required<br>1 Yes<br>0 No
	54	start_reintubation<br><br>Show the field ONLY if:<br>[re_intubated] = '1'	Start time of re-intubation	text (datetime_mdy), Required
	55	stop_reintubation<br><br>Show the field ONLY if:<br>[re_intubated] = '1'	Stop Time of re-intubation	text (datetime_mdy), Required
	56	time_reintubation<br><br>Show the field ONLY if:<br>[re_intubated] = '1'	Time of Re-Intubation (days)	calc, Required<br>Calculation: datediff([stop_reintubation], [start_reintubation],"d","mdy")
	57	re_intubated_third<br><br>Show the field ONLY if:<br>[re_intubated] = '1'	Was the patient re-intubated a third time during their hospital stay?	yesno, Required<br>1 Yes<br>0 No
	58	start_reintubation_2<br><br>Show the field ONLY if:<br>[re_intubated_third] = '1'	Start time of re-intubation	text (datetime_mdy), Required
	59	stop_reintubation_2<br><br>Show the field ONLY if:<br>[re_intubated_third] = '1'	Stop Time of re-intubation	text (datetime_mdy), Required
	60	time_reintubation_2<br><br>Show the field ONLY if:<br>[re_intubated_third] = '1'	Time of Re-Intubation (days)	calc, Required<br>Calculation: datediff([stop_reintubation_2], [start_reintubation_2],"d","mdy")
	61	noninv_mech_ventilation	Did the patient require non-invasive mechanical ventilation?	yesno, Required<br>1 Yes<br>0 No

	62	ed_noninv_mech_vent<br>Show the field ONLY if:<br>[noninv_mech_ventilation] = '1'	Was non-invasive mechanical ventilation initiated in the ED?	yesno, Required<br>1 Yes<br>0 No
	63	noninv_mech_vent_type<br>Show the field ONLY if:<br>[noninv_mech_ventilation] = '1'	What type of non-invasive mechanical ventilation did the patient receive? (check all that apply)	checkbox, Required<br>1 noninv_mech_vent_type___1 CPAP<br>2 noninv_mech_vent_type___2 Bipap<br>3 noninv_mech_vent_type___3 High-flow nasal O2
	64	start_noninvmech_vent<br>Show the field ONLY if:<br>[noninv_mech_ventilation] = '1'	Start Time of non-invasive mechanical ventilation	text (datetime_mdy), Required
	65	stop_noninvmech_vent<br>Show the field ONLY if:<br>[noninv_mech_ventilation] = '1'	Stop Time of non-invasive mechanical ventilation	text (datetime_mdy), Required
	66	time_noninvmech_vent<br>Show the field ONLY if:<br>[noninv_mech_ventilation] = '1'	Time of non-invasive mechanical ventilation (days)	calc, Required<br>Calculation: datediff([stop_noninvmech_vent], [start_noninvmech_vent],"d","mdy")
	67	tracheostomy	Did the patient have a tracheostomy while they were hospitalized?	yesno, Required<br>1 Yes<br>0 No
	68	tracheostomy_date<br>Show the field ONLY if:<br>[tracheostomy] = '1'	Tracheostomy Date	text (date_mdy), Required
	69	anticoag_prior	Section Header: *Anticoagulation- Complete this section at time of discharge*<br>Was the patient taking an anticoagulant prior to this visit (as a home med)?	yesno, Required<br>1 Yes<br>0 No
	70	anticoag_type_prior<br>Show the field ONLY if:<br>[anticoag_prior] = '1'	What anticoagulant were they taking at home?	radio, Required<br>1 eliquis/apixaban<br>2 xarelto/rivaroxiban<br>3 dabigatran/pradaxa<br>4 warfarin/coumadin<br>5 enoxaparin/lovenox
	71	anticoag_indication<br>Show the field ONLY if:<br>[anticoag_prior] = '1'	Indication for anticoagulation at home	checkbox, Required<br>1 anticoag_indication___1 atrial fibrillation<br>2 anticoag_indication___2 PE<br>3 anticoag_indication___3 DVT<br>4 anticoag_indication___4 stroke<br>5 anticoag_indication___5 mechanical heart valve<br>6 anticoag_indication___6 other
	72	anticoag_indication_other<br>Show the field ONLY if:<br>[anticoag_indication(6)] = '1'	If other, please specify	text, Required
	73	anticoagulants	Did the patient receive any medications for anticoagulation during their visit?	yesno, Required<br>1 Yes<br>0 No

	74	anticoagulation_type<br><br>Show the field ONLY if:<br>[anticoagulants] = '1'	What type of anticoagulation did the patient receive?<br><br>Prophylactic- heparin subcutaneous, enoxaparin/lovenox at a dose of 40 mg bid<br><br>Therapeutic- any heparin drip, eliquis/apixiban, xarelto/rivaroxiban, dabigatran/pradaxa, warfarin/coumadin, enoxaparin/lovenox at a dose of 1 mg/kg bid or 1.5 mg/kg qdaily	checkbox, Required<br>1 anticoagulation_type___1 prophylactic<br>2 anticoagulation_type___2 therapeutic
	75	anticoag_prophylactic<br><br>Show the field ONLY if:<br>[anticoagulation_type(1)] = '1'	What was given for prophylactic anticoagulation?	checkbox, Required<br>1 anticoag_prophylactic___1 heparin<br>2 anticoag_prophylactic___2 enoxaparin/lovenox
	76	anticoag_prophylactic_start<br><br>Show the field ONLY if:<br>[anticoagulation_type(1)] = '1'	Start date of prophylactic anticoagulation	text (date_mdy), Required
	77	anticoag_prophylactic_stop<br><br>Show the field ONLY if:<br>[anticoagulation_type(1)] = '1'	Stop Date of prophylactic anticoagulation	text (date_mdy), Required
	78	anticoag_therapeutic<br><br>Show the field ONLY if:<br>[anticoagulation_type(2)] = '1'	What was given for therapeutic anticoagulation?	checkbox, Required<br>1 anticoag_therapeutic___1 heparin<br>2 anticoag_therapeutic___2 eliquis/apixaban<br>3 anticoag_therapeutic___3 xarelto/rivaroxiban<br>4 anticoag_therapeutic___4 dabigatran/pradaxa<br>5 anticoag_therapeutic___5 warfarin/coumadin<br>6 anticoag_therapeutic___6 enoxaparin/lovenox
	79	anticoag_thera_start<br><br>Show the field ONLY if:<br>[anticoagulation_type(2)] = '1'	Start Date of Therapeutic Anticoagulation	text (date_mdy), Required
	80	anticoag_thera_stop<br><br>Show the field ONLY if:<br>[anticoagulation_type(2)] = '1'	Stop Date of Therapeutic Anticoagulation	text (date_mdy), Required
	81	steroids	Section Header: *Steroids- complete at time of discharge*<br>Was the patient given steroids during this visit?	yesno, Required<br>1 Yes<br>0 No
	82	steroid_type<br><br>Show the field ONLY if:<br>[steroids] = '1'	What steroid was the patient given?	checkbox, Required<br>1 steroid_type___1 prednisone<br>2 steroid_type___2 methylprednisolone/solumedrol<br>3 steroid_type___3 dexamethasone/decadron<br>4 steroid_type___4 hydrocortisone/soul-cortef
	83	start_steroids<br><br>Show the field ONLY if:<br>[steroids] = '1'	Start Date of Steroids	text (date_mdy), Required
	84	stop_steroids<br><br>Show the field ONLY if:<br>[steroids] = '1'	Stop Date of Steroids	text (date_mdy), Required
	85	treatments_complete	Section Header: *Form Status*<br>Complete?	dropdown<br>0 Incomplete<br>1 Unverified<br>2 Complete

Instrument: **Disposition And Outcomes** (disposition_and_outcomes) Collapse

	86	disposition	Disposition	radio, Required<br>1 Discharged Home<br>2 Regular Admission<br>3 ICU Admission<br>4 Expired in ED<br>5 Transferred to another hospital
	87	ed_diagnosis	Final ED Diagnosis	checkbox, Required<br>1 ed_diagnosis___1 COVID-19 Infection<br>2 ed_diagnosis___2 Respiratory Infection, not confirmed COVID<br>3 ed_diagnosis___3 SOB not specified<br>4 ed_diagnosis___4 Other
	88	ed_diagnosis_other<br>Show the field ONLY if:<br>[ed_diagnosis(4)] = '1'	If Other, please specify:	text, Required
	89	icu_upgrade<br>Show the field ONLY if:<br>[disposition] = '2'	Was the patient upgraded to the ICU during their hospitalization?	yesno, Required<br>1 Yes<br>0 No
	90	time_icu_upgrade<br>Show the field ONLY if:<br>[icu_upgrade] = '1'	Date and Time of ICU Upgrade	text (datetime_mdy), Required
	91	hosp_diagnosis<br>Show the field ONLY if:<br>[disposition] = '2' or [disposition] = '3'	Final Hospital Diagnosis	checkbox, Required<br>1 hosp_diagnosis___1 COVID-19 Infection<br>2 hosp_diagnosis___2 Respiratory Infection, not confirmed COVID<br>3 hosp_diagnosis___3 SOB not specified<br>4 hosp_diagnosis___4 Other
	92	hosp_diagnosis_other<br>Show the field ONLY if:<br>[hosp_diagnosis(4)] = '1'	If Other, please specify:	text, Required
	93	pulmonary_embolism	Was the patient diagnosed with a PE during this visit?	yesno, Required<br>1 Yes<br>0 No
	94	pe_date<br>Show the field ONLY if:<br>[pulmonary_embolism] = '1'	Date of pulmonary embolism diagnosis	text (date_mdy), Required
	95	dvt	Was the patient diagnosed with a DVT during this visit?	yesno, Required<br>1 Yes<br>0 No
	96	dvt_date<br>Show the field ONLY if:<br>[dvt] = '1'	Date of DVT diagnosis	text (date_mdy), Required
	97	limb_ischemia	Was the patient diagnosed with acute limb ischemia during this visit?	yesno, Required<br>1 Yes<br>0 No
	98	date_limb_ischemia<br>Show the field ONLY if:<br>[limb_ischemia] = '1'	Date of acute limb ischemia diagnosis	text (date_mdy), Required

	99	mesenteric_ischemia	Was the patient diagnosed with mesenteric ischemia during this visit?	yesno, Required<br>1 Yes<br>0 No
	100	mesenteric_ischemia_date<br>Show the field ONLY if: [mesenteric_ischemia] = '1'	Date of mesenteric ischemia diagnosis	text (date_mdy), Required
	101	stroke	Was the patient diagnosed with a stroke during this visit?	yesno, Required<br>1 Yes<br>0 No
	102	stroke_date<br>Show the field ONLY if: [stroke] = '1'	Date of stroke diagnosis	text (date_mdy)
	103	mi	Was the patient diagnosed with a myocardial infarction during this visit?	yesno, Required<br>1 Yes<br>0 No
	104	mi_date<br>Show the field ONLY if: [mi] = '1'	Date of MI diagnosis	text (date_mdy)
	105	mi_type<br>Show the field ONLY if: [mi] = '1'	Type of MI	radio, Required<br>1 Type I<br>2 Type II<br>3 Unknown
	106	otherclottingevent	Was the patient diagnosed with any other type of clotting event?	yesno, Required<br>1 Yes<br>0 No
	107	otherclottingevent_date<br>Show the field ONLY if: [otherclottingevent] = '1'	Date of other clotting event	text (date_mdy), Required
	108	otherclottingevent_specify<br>Show the field ONLY if: [otherclottingevent] = '1'	Specify type of other clotting event	text
	109	quarantine	At discharge, was the patient told to self-quarantine?	yesno, Required<br>1 Yes<br>0 No
	110	death	Did patient die during this encounter?	yesno, Required<br>1 Yes<br>0 No
	111	code_status	Patient code status (check at discharge)	radio, Required<br>1 Full Code<br>2 DNR<br>3 DNR/DNI<br>4 comfort care
	112	disposition_and_outcomes_complete	Section Header: *Form Status*<br>Complete?	dropdown<br>0 Incomplete<br>1 Unverified<br>2 Complete
Instrument: **Followup** (followup)				Collapse
	113	return_subjectid	Subject Number of Return Visit #1 in Redcap	text, Required
	114	return_subjectid_2	Subject Number of Return Visit #2 in Redcap	text

	115	return_subjectid_3	Subject Number of Return Visit #3 in Redcap	text
	116	return_subjectid_4	Subject Number of Return Visit #4 in Redcap	text
	117	followup_complete	Section Header: *Form Status*<br>Complete?	dropdown<br>0 Incomplete<br>1 Unverified<br>2 Complete
